# Supplementary material for: Empirical methods for controlling false positives and estimating confidence in ChIP-Seq peaks
Source: BMC Bioinformatics. 2008 Dec 5;9:523. doi: 10.1186/1471-2105-9-523 (PMC2628906; doi:10.1186/1471-2105-9-523)
Supplement: Additional File 1 — A variety of html documents from the USeq web site detailing the available applications, their best usage, output file type descriptions, command line menus, etc. [file 1471-2105-9-523-S1.zip › USeqUserGuides/JavaDoc.zip!/JavaDoc/edu/utah/seq/data/EnrichedRegion.html]

EnrichedRegion


|  |  |  |
| --- | --- | --- |
| **Overview** \| **Package** \| **Class** \| **Use** \| **Tree** \| **Deprecated** \| **Index** \| **Help** | |  |
| **PREV CLASS**   **NEXT CLASS** | **FRAMES**    **NO FRAMES**     **All Classes** |  |
| SUMMARY: NESTED \| FIELD \| CONSTR \| METHOD | DETAIL: FIELD \| CONSTR \| METHOD |  |


---


## edu.utah.seq.data Class EnrichedRegion

```
java.lang.Object
  edu.utah.seq.data.EnrichedRegion
```

**All Implemented Interfaces:**: java.io.Serializable

---

``` public class EnrichedRegion extends java.lang.Object implements java.io.Serializable ```

**See Also:**: Serialized Form

---

| **Field Summary** | |
| --- | --- |
| `static long` | `serialVersionUID` |


**Constructor Summary**

`EnrichedRegion()`

`EnrichedRegion(SmoothingWindow window,
Info info)`

`EnrichedRegion(SmoothingWindow window,
int halfReadLength)`


| **Method Summary** | |
| --- | --- |
| `SmoothingWindow` | `getBestSubWindow()` |
| `SmoothingWindow` | `getBestWindow()` |
| `java.lang.String` | `getChromosome()` |
| `Point[]` | `getControlPoints()` |
| `int` | `getNumberOfWindows()` |
| `java.lang.String` | `getSequence()` |
| `int` | `getStart()` |
| `int` | `getStop()` |
| `SmoothingWindow[]` | `getSubWindows()` |
| `Point[]` | `getTreatmentPoints()` |
| `void` | `setBestSubWindow(SmoothingWindow bestSubWindow)` |
| `void` | `setBestWindow(SmoothingWindow bestWindow)` |
| `void` | `setChromosome(java.lang.String chromosome)` |
| `void` | `setControlPoints(Point[] controlPoints)` |
| `void` | `setNumberOfWindows(int numberOfWindows)` |
| `void` | `setSequence(java.lang.String sequence)` |
| `void` | `setStart(int start)` |
| `void` | `setStop(int stop)` |
| `void` | `setSubWindows(SmoothingWindow[] subWindows)` |
| `void` | `setTreatmentPoints(Point[] treatmentPoints)` |

**Methods inherited from class java.lang.Object**

`equals, getClass, hashCode, notify, notifyAll, toString, wait, wait, wait`

**Field Detail**

### serialVersionUID

```
public static final long serialVersionUID
```

**See Also:**: Constant Field Values


**Constructor Detail**

### EnrichedRegion

```
public EnrichedRegion(SmoothingWindow window,
                      Info info)
```

---


### EnrichedRegion

```
public EnrichedRegion(SmoothingWindow window,
                      int halfReadLength)
```

---


### EnrichedRegion

```
public EnrichedRegion()
```


**Method Detail**

### getBestWindow

```
public SmoothingWindow getBestWindow()
```

---


### setBestWindow

```
public void setBestWindow(SmoothingWindow bestWindow)
```

---


### getChromosome

```
public java.lang.String getChromosome()
```

---


### setChromosome

```
public void setChromosome(java.lang.String chromosome)
```

---


### getNumberOfWindows

```
public int getNumberOfWindows()
```

---


### setNumberOfWindows

```
public void setNumberOfWindows(int numberOfWindows)
```

---


### getSequence

```
public java.lang.String getSequence()
```

---


### setSequence

```
public void setSequence(java.lang.String sequence)
```

---


### getStart

```
public int getStart()
```

---


### setStart

```
public void setStart(int start)
```

---


### getStop

```
public int getStop()
```

---


### setStop

```
public void setStop(int stop)
```

---


### getControlPoints

```
public Point[] getControlPoints()
```

---


### setControlPoints

```
public void setControlPoints(Point[] controlPoints)
```

---


### getTreatmentPoints

```
public Point[] getTreatmentPoints()
```

---


### setTreatmentPoints

```
public void setTreatmentPoints(Point[] treatmentPoints)
```

---


### getSubWindows

```
public SmoothingWindow[] getSubWindows()
```

---


### setSubWindows

```
public void setSubWindows(SmoothingWindow[] subWindows)
```

---


### getBestSubWindow

```
public SmoothingWindow getBestSubWindow()
```

---


### setBestSubWindow

```
public void setBestSubWindow(SmoothingWindow bestSubWindow)
```


---


|  |  |  |
| --- | --- | --- |
| **Overview** \| **Package** \| **Class** \| **Use** \| **Tree** \| **Deprecated** \| **Index** \| **Help** | |  |
| **PREV CLASS**   **NEXT CLASS** | **FRAMES**    **NO FRAMES**     **All Classes** |  |
| SUMMARY: NESTED \| FIELD \| CONSTR \| METHOD | DETAIL: FIELD \| CONSTR \| METHOD |  |


---
